# An Imperative for the National Public Health School in Burkina Faso to Promote the Use of Information and Communication Technologies in Education During the COVID-19 Pandemic: Critical Analysis

**DOI:** 10.2196/27169

**Published:** 2021-05-18

**Authors:** Arzouma Hermann Pilabré, Patrice Ngangue, Abibata Barro, Yacouba Pafadnam

**Affiliations:** 1 Institut de Formation et de Recherche Interdisciplinaires en Sciences de la Santé et de l’Éducation Ouagadougou Burkina Faso; 2 Faculté de médecine et sciences de la santé Université de Sherbrooke Sherbrooke, QC Canada

**Keywords:** Burkina Faso, teaching, learning, ICT, COVID-19, critical analysis, public health, online learning, e-learning, information and communication technology, challenge

## Abstract

**Background:**

Several studies have reported the positive impact of information and communication technologies (ICTs) on academic performance and outcomes. Although some equipment is available, the ICTs for education at the National Public Health School (NPHS) of Burkina Faso have many shortcomings. These shortcomings were clearly revealed during the search for responses to the crisis caused by the COVID-19 pandemic. Indeed, to curb the spread of COVID-19, some measures were taken, such as closure of educational institutions. This resulted in a 2.5-month suspension of educational activities. Despite its willingness, the NPHS was unable to use ICTs to continue teaching during the closure period of educational institutions.

**Objective:**

In this paper, we aim to propose practical solutions to promote ICT use in teaching at the NPHS by analyzing the weaknesses and challenges related to its use.

**Methods:**

We conducted a critical analysis based on information from the gray literature of NPHS. This critical analysis was preceded by a review of systematic reviews on barriers and facilitating factors to using ICTs in higher education and a systematic review of ICT use during the COVID-19 pandemic in higher education. An ICT integration model and a clustering of ICT integration factors guided the analysis.

**Results:**

The weaknesses and challenges identified relate to the infrastructure and equipment for the use of ICTs in pedagogical situations in face-to-face and distance learning; training of actors, namely the teachers and students; availability of qualified resource persons and adequate and specific financial resources; motivation of teachers; and stage of use of ICTs.

**Conclusions:**

To promote the use of ICTs in teaching at the NPHS, actions must be performed to strengthen the infrastructure and equipment, human resources, the skills of actors and the motivation of teachers in the pedagogical use of ICTs.

## Introduction

The rapid evolution of information and communication technologies (ICTs) has led to the development of applications for use in everyday life and in all activity sectors [1]. Faced with this development, the integration of ICTs has become a necessity in education systems [2]. In Burkina Faso, the National Public Health School (NPHS) began integrating and promoting ICTs in education approximately 10 years ago. This integration has resulted in the establishment of infrastructures and training of actors [3].

Located within West Africa, the country of Burkina Faso covers an area of 274,200 km^2^. It is subdivided into 13 regions, 45 provinces, 350 departments, and 351 municipalities [4]. The number of students per 100,000 inhabitants has increased from 336 in 2009-2010 to 600 in 2017-2018. Under the Education Guidance Act, the education system in Burkina Faso is organized into formal, nonformal, informal, and special education [5].

Since the 1980s, numerous private and public actions have been implemented to integrate ICTs in education in Burkina Faso [6]. The development of skills and abilities for the widespread use of ICTs is one of the challenges faced by the higher education system in Burkina Faso [4].

The NPHS is ranked in the Higher School category, which is a component of higher education. Its main mission is to ensure training of midwives and paramedical staff in primary and specialized fields to benefit the public and the private sector. The NPHS is organized as follows: the Board of Directors, which holds the highest administrative responsibility; and the Executive Board, which directs and coordinates all institution activities. The Executive Board includes the central and regional directorates. There are 10 regional directorates. In addition to the regional directorates, the Directorate of Higher Education in Health Science (DHEHS) is responsible for specialized training of paramedical and midwifery personnel. Each regional directorate and the DHEHS has the following work stations: a secretariat; a pedagogical service; training services; a school life service; two control rooms; and an administrative and financial service [3].

In 2006, the West African Health Organization, together with its member countries, including Burkina Faso, initiated harmonization of curricula. This harmonization, which adopted the Bachelor-Master-Doctorate (BMD) system in the Economic Community of West African States (ECOWAS), is seen as a means of regulating the training and career development of health professionals [7]. The harmonization began with the curricula for nurses and midwives, which were approved and adopted in 2010 by ECOWAS Health Ministers. From 2011, the NPHS entered into this process of harmonizing basic and postbasic training curricula. It then embarked on implementing the BMD system, starting with the nursing and midwifery streams. In the institution’s progression toward effective application of the BMD system, ICTs are of paramount importance. In this sense, the NPHS has equipped itself with a videoconferencing system installed in all the regional directorates except in the recently established ones of Dédougou, Ziniaré, and Banfora. This system enables video conferencing and distance learning to benefit the institution's trainers [3].

In the absence of a strategy document, it is not easy to obtain a clear picture of the design and process for implementing ICTs in teaching at the NPHS. Literature reports show that the use or integration of ICTs in education requires policy or strategies [8]. Pedagogical integration or use of ICTs in teaching refers not only to the educational institution equipment and networking but also to the appropriate, usual, and regular use of ICTs by teachers and students to support and enhance teaching and learning [9]. The use of ICTs in teaching can occur in a face-to-face educational situation and/or in a distance pedagogical situation in synchronous and/or asynchronous mode [10-27].

The shift to distance education can help institutions cope with unexpected situations, such as those caused by the COVID-19 pandemic. Indeed, due to the COVID-19 pandemic, most universities have moved to web-based distance learning in synchronous and/or asynchronous environments [10-27]. Several countries, including Burkina Faso, have imposed closure of educational and training institutions to ensure the respecting of physical distancing measures and to reduce the risk of contamination [10-27]. Although in some countries, this situation has led several educational structures to optimize the use of the potential of ICTs to provide e-learning to students, this has not been possible at the NPHS [28].

NPHS officials were unable to maintain teaching continuity due to inadequate and obsolete equipment [29] and poor preparation. This suspension of educational activities has had many consequences for students, teachers, and NPHS officials. Given the magnitude of these consequences, upgrading and promoting the effective use of ICT in education is becoming imperative for the NPHS, especially in the case of a second wave. This crisis also creates the opportunity for all systems to look to the future, adapt to possible threats, and strengthen their capacity [30].

The goal of this paper is to enable the NPHS and educational structures that are in a similar situation to exploit the potential offered by ICTs, through proposals for solutions, to improve the quality of training and to be able to address unexpected situations such as those generated by the COVID-19 pandemic.

## Methods

To perform the critical analysis, we first carried out two rapid systematic reviews. The methodology followed PRISMA-P (Preferred Reporting Items for Systematic Review and Meta-Analysis Protocols) [31]. The first review was a review of systematic reviews. Systematic reviews published between 2017 and 2021 that examined encountered difficulties in ICT use in higher education and strategies to overcome these difficulties were included. Systematic reviews on ICT use in primary or secondary schools or on individual courses or specific aspects such as gender were excluded. We searched three electronic bibliographic databases (ERIC, CINAHL, and PubMed) to identify systematic reviews focused on barriers and facilitators in using ICT in higher education. We used the following terms to develop the search strategies: *students*, *learners*, *teachers*, *trainers*, *educators*, *manager*, *higher education*, *university*, *information and communication technologies for education*, *ICT for education*, *web-based learning*, *e-learning*, *distance education*, *computerized technological resources*, *online learning*, *virtual classroom*, *virtual class*, *remote education*, *remote instruction*, *internet use for education*, *access to ICT*, *use of ICT*, *the capacity of use*, *perceived usefulness*, *barriers*, *facilitating factors*, and *systematic review*.

The search strategy for the PubMed database was as follows: (“Students” [MeSH] OR “Learners” OR “Teachers” OR “Trainers” OR “Campus managers” OR “Directors” OR “Education, Graduate” [MeSH] OR “Universities” [MeSH] OR “Faculty” [MeSH]) **AND** (“Information and communication technologies for education” OR “ICT for education” OR “Web-based learning” OR “E-learning” OR “Distance education” OR “Distance Learning” [MeSH] OR “Learning, Distance” [MeSH] OR “Computerized technological resources” OR “Online Learning” [MeSH] OR “Learning, Online” [MeSH] OR “Online Education” [MeSH] OR “Remote Education” OR “Remote instruction” OR “Virtual classes” OR “Virtual classroom” OR “Integration of ICT” OR “ICT” OR “Internet use” [MeSH] OR “Computer User Training” [MeSH]) **AND** (“Access to ICT” OR “Use of ICT” OR “Capacity of use” OR “Perceived usefulness” OR “Barriers” OR “Facilitating factors”). This strategy was adapted for use in the ERIC and CINAHL bibliographic databases.

**Figure 1:**
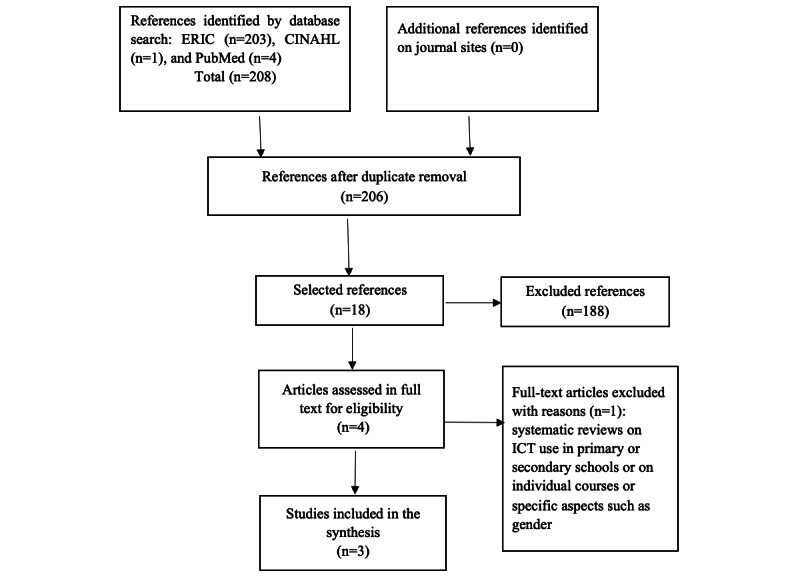
Adapted PRISMA (Preferred Reporting Items for Systematic Review and Meta-Analysis Protocols) flow diagram to show the results of the searches in the first literature review. ICT: information and communications technology.

Figure 1 illustrates the study selection process for the first literature review.

In the second literature review, we included published articles from 2020 to 2021 with primary data describing the use of ICTs during the COVID-19 pandemic in universities, faculties, and colleges. We excluded editorials, commentaries, and articles reporting experiences with web-based distance education and learning of specific courses, implementation projects, or web-based distance education evaluations. For this purpose, we searched three databases (ERIC, CINAHL, and PubMed). The following terms were used to develop the research strategies: *students*, *learners*, *teachers*, *trainers*, *educators*, *manager*, *higher education*, *university*, *COVID-19*, *information and communication technologies for education*, *ICT for education*, *web-based learning*, *e-learning*, *distance education*, *computerized technological resources*, *online learning*, *virtual classroom*, *virtual class*, *remote education*, *remote instruction*, *internet use for education*, *access to ICT*, *use of ICT*, *the capacity of use*, *perceived usefulness*, *confirmation of expectations*, *students' satisfaction*, *knowledge*, *attitudes*, *practice*, and *students' engagement*.

The search strategy for the PubMed database was as follows: (“Students” [MeSH] OR “Learners” OR “Teachers” OR “Trainers” OR “Campus managers” OR “Directors” OR “Education, Graduate” [MeSH] OR “Universities” [MeSH] OR “Faculty” [MeSH] OR “COVID-19” [MeSH]) **AND** (“Information and communication technologies for education” OR “ICT for education” OR “Web-based learning” OR “E-learning” OR “Distance education” OR “Distance Learning” [MeSH] OR “Learning, Distance” [MeSH] OR “Computerized technological resources” OR “Online Learning” [MeSH] OR “Learning, Online” [MeSH] OR “Online Education” [MeSH] OR “Remote Education” OR “Remote instruction” OR “Virtual classes” OR “Virtual classroom” OR “Integration of ICT” OR “ICT” OR “Internet use” [MeSH] OR “Computer User Training” [MeSH]) **AND** (“Access to ICT” OR “Use of ICT” OR “Capacity of use” OR “Perceived usefulness” OR “Confirmation of expectations” OR “Student satisfaction” OR “Health knowledge, attitudes, practice” OR “Health Knowledge, Attitudes, Practice” [MeSH] OR “Student engagement” OR “Academic Success” [MeSH] OR “Learning” OR “Professional Competence” [MeSH] OR “mental competency” [MeSH] OR “Skills”). This strategy was adapted for use in the ERIC and CINAHL bibliographic databases.

**Figure 2:**
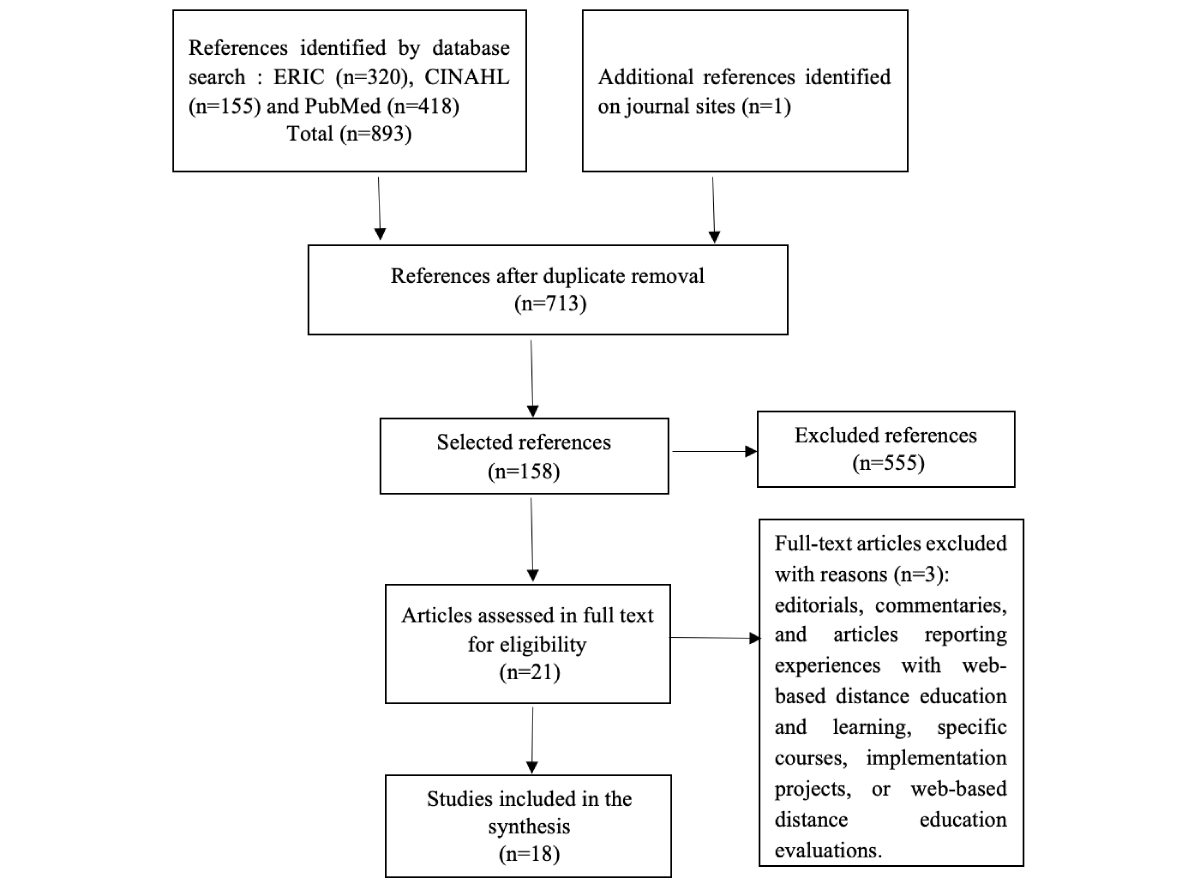
Adapted PRISMA (Preferred Reporting Items for Systematic Review and Meta-Analysis Protocols) flow diagram to show the results of the searches in the second literature review.

Figure 2 illustrates the study selection process for the second literature review.

The database search results were stored in a single reference manager software (Zotero). Duplicate references were removed. Titles and abstracts of the review papers retrieved using the search strategy were screened.

A standardized data extraction form was developed, piloted, and used to extract data from the full text of the included publications. In addition to the general characteristics of the studies, we extracted data regarding the use of ICTs in teaching, learning, and the management of the COVID-19 pandemic in high schools.

An ICT integration model and a clustering type of ICT integration factors guided the data synthesis. The information concerning the NPHS was taken from the gray literature of the institution.

## Results

### Literature Reviews

In the first systematic review on barriers and facilitators of ICT use in higher education, a search of the three databases identified 208 articles. We deemed 3 articles to be relevant. The articles included are those by Webb et al [8], Regmi et al [32], and Atmacasoy et al [33]; the selected systematic reviews date from 2017, 2018, and 2020, respectively. Of these reviews, 2 were conducted in the United Kingdom [8,32] and 1 in Turkey [33]. The 3 systematic reviews included 128 articles and 10 theses [8,32,33].

For the second systematic review on the use of ICTs in higher education during the COVID-19 pandemic, 893 articles were retrieved from the databases. The search of websites of specialized journals yielded 1 additional article, for a total of 894 articles. We deemed 18 articles to be relevant. The articles included are those of van der Keylen et al [10], Soy-Muner [11], Daniel [12], Moszkowicz et al [13], Yılmaz et al [14], Al-Balas et al [15], Sharma [16], George [17], Kim et al [18], Sabharwal et al [19], Sutiah et al [20], Scull et al [21], Barik et al [22], Khalaf [23], Mansoor [24], Ibrahim et al [25], Lowenthal et al [26], and Chick et al [27]. Of these 18 articles, 8 are from Asia, 4 from America, 4 from Europe, 1 from Africa, and 1 from Oceania. All these articles were published in 2020. Most of the studies first presented a section that describes the use of ICTs during the COVID-19 pandemic and another section devoted to assessment.

### Web-Based Distance Education in Higher Education

ICTs are used in higher education to achieve web-based distance education and learning. The blended learning mode is the most widely used. A systematic review [32], which included 21 articles and 10 theses, reported that most web-based distance education studies focused on a blended learning environment via Moodle. Moodle is a free learning management system for creating flexible and engaging web-based experiences or a website specifically designed for a blended learning course. Blended learning is defined as a combination of learning delivery methods, including face-to-face teaching with asynchronous or synchronous computer technologies [32]. Some of the descriptions of the components of blended learning are as follows [32]:

- Carious web technology tools are combined, such as live virtual classrooms, collaborative learning and streaming video.
- An optimal learning outcome is achieved with or without instructional technology by combining different pedagogical approaches, such as constructivism, behaviorism, and cognitivism.
- Any form of instructional technology (eg, videotape, CD-ROM, e-learning, and film) is combined with face-to-face instruction.
- Instructional technology is combined with real-world tasks to support work-based learning.

Blended learning has brought several benefits, mainly due to the successful merging of face-to-face and web-based aspects by making resources more accessible. It promotes the student-centered approach by providing various materials, increasing participation, and fostering student-student and teacher-student interaction. In addition, it provides timely feedback and creates a ground for synchronous and asynchronous discussions [32].

### Encountered Difficulties in Web-Based Distance Learning

Encountered difficulties in web-based distance education in higher education are related to personal, institutional, and pedagogical factors.

#### Personal Factors

Personal factors relate to teachers and students' motivation and commitment to using ICTs in teaching and learning [34].

One of the reported personal factors is teacher anxiety due to the considerable importance of using ICTs in blended learning [32]. Students also have high levels of anxiety and stress related to the use of ICTs in learning. These high levels of anxiety and stress are due to inappropriate equipment and technological illiteracy [8].

Another difficulty related to personal factors is low motivation or lack of enthusiasm of teachers and students for educational technology [8,33]. Low motivation about web-based distance education refers to low commitment, poor perception, limited flexibility, lack of student self-discipline, low self-efficacy, and poor interaction between learners and facilitators [8].

#### Institutional Factors

Institutional factors include creating an adequate pedagogical environment that enables teachers to apply ICT in teaching methods [34].

A systematic review has highlighted some of the barriers that threaten the construction of effective blended learning environments. These barriers include infrastructure problems, connection failures and slow internet access, technical problems, and lack of personal computers [32,33]. Lack of internal support for ICT use is also a concern for both students and faculty [33].

In one review, 9 out of 24 articles reported that e-learning is a time-, cost-, and labor-intensive approach. Insufficient resources are a significant barrier. A total of 8 out of 24 articles identified the lack of a computer or user-friendly computer as one of the main challenges to successful e-learning [8].

It was also pointed out that problems related to cost and availability of resources in the long term raise concerns for ensuring quality, user-friendliness, and distance education and learning effectiveness. In addition, insufficient consideration of users' needs and lack of time are barriers that will negatively impact e-learning [8].

#### Pedagogical Factors

Pedagogical factors take into account the technical abilities of teachers to use a computer. To this end, teachers must design teaching materials and produce courses with multimedia support to support and facilitate student learning [34].

The most frequently encountered barriers are lack of teachers' computer skills [33], poor course structure, poor instructional design, absence of clear objectives, limited use of technology in teaching, and insufficient teacher training [8]. Indeed, the university staff is also concerned about the lack of training and time needed to develop asynchronous learning regimes and invest more ICT resources in their teaching [33]. At the learner level, several articles also raised technological or computer challenges. Indeed, many learners are not familiar with e-learning, and in some contexts, they even lack basic computer literacy [8].

Another obstacle identified is related to the fact that web-based distance learning is not suitable for all disciplines or contents. A total of 8 of 24 papers reported that integrating learning into existing programs would be problematic, as some disciplines would take a long time for learners and facilitators to adapt the content in e-learning programs. Moreover, several articles reported that some content may be unsuitable for e-learning, but some content may not be appropriate because these disciplines need practical or demonstrative types of learning [8].

### Strategies to Overcome the Difficulties Encountered in Web-Based Distance Education

To overcome the difficulties encountered in web-based distance education, the development of appropriate institutional strategies is essential. These institutional strategies could include flexibility of web-based distance education, access to systems, costs, learning styles, training of teachers and learners, and exploitation of local systems management of learning [8].

In addition, human and environmental barriers such as beliefs and motivation of staff and students must be overcome. Substantial financial resources must be mobilized to finance the long-term functioning of web-based distance education and learning systems. Furthermore, faculties or universities should allow time for training of teachers and students and for course content preparation. They should also provide technical support staff and effective systems for web-based distance education [33].

### ICT Use During the COVID-19 Pandemic

The closure of educational institutions caused by the COVID-19 pandemic encourages optimal exploitation of the potential offered by ICTs around the world [10-27]. ICT has been used primarily to provide distance education and learning on the web. All of the studies included in this systematic review described using ICT in universities during the COVID-19 pandemic to provide distance teaching and learning or education on the web [10-27].

Most studies have reported that the synchronous and asynchronous use of web-based distance teaching and learning is the option chosen by universities [10-13], [16,17], [19,20], [23-27]. This choice could be explained by the fact that web-based learning works best when the material designed, used asynchronously by students, is associated with synchronous class discussions [12]. Teaching synchronous and asynchronous learning consists of live lectures and pre-recorded lectures or SMS text messages made available to students [10-13], [16,17], [19,20], [24-27]. The videoconferencing method can be applied to clinical lessons and anatomy lessons [13].

A total of 2 studies described the option provided by universities to realize web-based distance learning and teaching in synchronous form. This uniquely synchronous web-based distance learning occurs through live teleconferences or webinars and through educational meetings held on different web platforms [18,21].

Only one study reported web-based distance learning education by a university in the asynchronous form through video applications. The option of the exclusively asynchronous form was made due to constraints following the synchronous form [25].

A useful resource in face-to-face teaching restrictions is that of a very detailed workbook-type text. The text presents elements for all of the course topics using step-by-step solutions to problems and diagrams. Practical questions and their answers are presented at the end of each chapter. This resource is made available to students for download [16].

Beyond lessons, ICTs have been used to conduct examinations or train students by remote evaluations [16,22,24]. An app is used in combination with a browser for written examinations. Oral examinations are organized as web-based meetings [22]. Simulated web-based quizzes are also sent to students to enable them to answer structured questions and to familiarize them with the web-based examinations [16].

To be effective, adoption of early web-based distance education and learning by universities must meet certain conditions. Comprehensive web-based teaching and learning require rich lesson plan design and quality and engaging instructional content supported by audio and video content with strong technology support teams. The smooth migration to web-based teaching and learning requires the implementation of an educational policy of (1) grouping and reorganizing course content into smaller, more understandable units to help students navigate, focus, and understand; (2) emphasizing the use of “modulation, inflexion, pitch and timbre of the voice” in web-based education; (3) training the faculty, because the technical specifications of web-based education are much higher than those of traditional classroom instruction for inexperienced faculty members who deliver educational content on the web for the first time; (4) reinforcing students' active learning skills, as compared to traditional lessons, teachers have less control over web-based instruction, and students are more likely to avoid lessons; (5) developing the concept of web-based and offline self-learning [27].

## Discussion

### Principal Findings and Recommendations

The NPHS should exploit the potential of ICTs to avoid the total suspension of educational activities for approximately 2.5 months. Early leaders thought about this but soon encountered the limitations of using ICTs in teaching in their institution. It is this suspension of educational activities at NPHS for a long time during the COVID-19 pandemic that motivated this critical analysis.

The results of the review of systematic reviews indicate that ICTs have long been used in higher education in blended learning modalities [32]. Difficulties are encountered in web-based distance learning. These difficulties include the anxiety and lack of motivation of teachers and students, insufficient pedagogical and teachers' computer skills, insufficient connection to the internet, lack of time for teachers, insufficient infrastructure and equipment, insufficient human and financial resources, and insufficient computer skills among students [8,32,33]. Solutions to overcome these difficulties have been suggested. These solutions involve developing appropriate institutional strategies, the motivation of the main actors, the mobilization of financial resources, and the strengthening of infrastructure and equipment [8,33]. The systematic review shows that the use of ICTs in higher education has intensified and spread with the advent of the COVID-19 pandemic. Several universities or faculties have moved to web-based distance education and learning in a synchronous or asynchronous environment [10-27]. One of the difficulties of using ICT in higher education linked to personal factors is low motivation or lack of enthusiasm for educational technology teachers and students [8,33]. The integration of ICTs is an innovation whose application requires the motivation of teachers [33]. The NPHS also encounters this difficulty because the motivation of teachers to use ICT is nonexistent. The evaluation of lessons that could encourage, value, and reward teachers is not implemented [3].

To remedy teachers' lack of motivation to use ICTs effectively [33], the authors recommend that the NPHS develop strategies to recognize and value the teaching profession using ICTs. One strategy could be course evaluation followed by rewards for the best teachers. In addition, teachers’ involvement in decision-making concerning ICT use in education must be strengthened because it is also a motivating factor [33]. The obstacles that threaten the construction of effective blended learning environments include infrastructure problems, connection failures and slow internet access, technical problems, and a lack of personal computers [32,33]. In short, there is no conducive educational environment for teachers to apply ICT to teaching techniques. The educational environment should be accompanied by equipment of teachers with technopedagogical tools, the establishment of adequate infrastructure and equipment, and the establishment and training of teachers and students in the educational applications of ICTs. A favorable educational environment requires the creation of a structure that is responsible for the educational integration of ICTs to provide leadership to general or regional management [35].

At the NPHS, teachers do not have computers or accessories such as USB keys, servers, cables, connection wires, telecommunications links, videoconferencing equipment, and networks or operating software [3] to enable the educational integration of ICTs in their professional practice. Pending the development and implementation of a specific plan to respond to the lack of infrastructure and equipment and the poor access to a fluid and permanent internet connection, the authors of the article recommend that the NPHS build infrastructures and equip the regional offices with distance education facilities, high-speed internet access systems, and other ICT equipment of sufficient quantity and quality [33]. These investments can be made through advocacy with the Ministry of Health and technical and financial partners. In addition, the NPHS must facilitate the acquisition of computer and pericomputer equipment by students and teachers. Students’ acquisition of computer equipment could be facilitated by pleading with the president of Burkina Faso for the inclusion of NPHS students in the “one student, one computer” program. This program aims to provide each participating student with a computer at a subsidized price. In fact, a study showed that the “one student, one computer” program was effective [36]. A special operation focusing on flexible payment terms could be organized to provide permanent teachers with computers. It has also been pointed out that issues related to the cost and availability of long-term resources raise concerns to ensure quality, usability, distance education, and learning efficiency [8]. The availability of substantial financial resources is essential to ensure the permanent functioning of ICTs and address the costs of maintenance and renewal of technological equipment. Fundraising or providing adequate, equitable, and stable funding is essential to acquire technological resources [37]. At the NPHS, adequate and specific financial resources for using ICTs in education are not available [3]. The administration of the NPHS and active help from partners and parents can help subsidize the internet subscription and the ICT equipment [37]. Technological infrastructure requires regular and consistent funding, mainly because of the rapid pace of technological change [38]. In addition, ICT equipment is not regularly renewed due to a lack of funding. For example, none of the 23 initial computers in the computer room of the regional office of the NPHS in Ouagadougou is currently functional [39]. In this regional office, it is impossible to access the internet connection despite the installation of modems [39]. To obtain financial resources for the maintenance of ICT equipment and to ensure a permanent subscription to an internet connection and the renewal of ICT equipment [33,38], the authors of the article advise the NPHS to dedicate a specific budget line to this objective each year in its action plan [33].

The lack of internal support in terms of specialized human resources for ICT use is also a concern for students and teachers [33]. The availability of qualified resource persons such as an information technology (IT) specialist, a trainer, a tutor or an instructor to provide support and training in ICT to teachers is insufficient [33]. These professionals provide the necessary technical support to students and teachers [33]. Their technical assistance role can facilitate, among other things, research, the creation of a resource bank for teachers and students, and the safe use of equipment [37]. According to some authors, to fully exploit technology, four human resources categories are necessary: ​​technical support staff; media production and management staff; instructional designers; and finally, teachers, professors, or content creators [38].

At the NPHS, this type of staff does not exist in any regional directorate. The only IT specialist recruited, who can be considered as a technical assistant, is assigned to general management [3]. Faced with the lack of human resources, the authors of the article recommend that the general management of the NPHS recruit and make available to the regional offices the necessary resource persons to promote the use of ICTs in education [33]. It would also be advantageous for the NPHS to develop partnerships with training establishments or universities with ICT experience related to education.

The most frequently encountered obstacles are the lack of computer skills of teachers [33], poor course structure, poor instructional design, lack of clarity of objectives, limited use of technology in teaching, and inadequate and insufficient training of teachers [8]. The establishment of adequate infrastructure and equipment must be accompanied by training of teachers and students in the pedagogical applications of ICTs. Teachers must be able to produce teaching materials and lessons with multimedia support to facilitate student learning [35]. No adequate training on the use of ICTs in education has been organized for teachers [8]. This lack of training is not conducive to effective and efficient pedagogical use of ICTs.

The majority of NPHS teachers cannot design teaching materials and produce courses with multimedia support to support and facilitate student learning. One of the manifestations of this lack of skills is the lack of educational innovation [3]. To improve teachers’ ability to reach the stage of pedagogical use of ICTs [9] in teaching, the authors recommend that the NPHS organize training sessions for these teachers [33]. These training sessions should aim to make teachers capable of producing teaching material and multimedia support courses [34]. In addition, teachers must be made aware of the need for self-training. The stage of “pedagogical use” of ICTs begins when the teacher feels a pedagogical curiosity, need, or obligation [9].

All the articles included in the systematic review on the use of ICTs in universities during the COVID-19 pandemic showed that ICTs were used in these settings to ensure distance teaching and learning [10-27]. Only the use of ICTs could offer the possibility for universities to maintain contact with students and to continue certain educational activities during the closure of educational institutions to contribute to the reduction of the spread of the pandemic of COVID-19 [28]. However, the authors of the included articles did not explicitly present the methodology that was employed to describe this use of ICTs [10-27].

The unexpected closure of the NPHS, which resulted in the suspension of educational activities for a long time, had many negative consequences. The NPHS should exploit the potential of ICTs to avoid the total suspension of educational activities for approximately 2.5 months. Early leaders thought about this but soon came up against the limitations of using ICTs in teaching in their institution.

Most studies have reported that the synchronous and asynchronous use of web-based distance teaching and learning is the option chosen by universities [10-13,16,17,19,20,23-27]. This choice could be explained by the fact that web-based learning works best when the material designed to be used by students asynchronously is associated with synchronous class discussions [12]. To begin web-based distance education and learning, the NPHS could opt for the asynchronous form because the synchronous form has many more constraints [25]. This asynchronous use could be achieved by providing students with prerecorded lectures, PowerPoint presentations, or detailed SMS text messages [10-13,16].

To overcome the difficulties encountered in web-based distance education, the development of appropriate institutional strategies is essential [8]. These institutional strategies could include the flexibility of web-based distance education, access to systems, costs, learning styles, training of teachers and learners, and exploitation of local systems management of learning [8]. The implementation of conditions for integrating ICTs in education must be preceded by developing specific policies, strategies, or plans that take this aspect into account [11].

The NPHS does not have a policy document on the integration or use of ICTs in education [3]. In 2019, the NPHS adopted a Strategic Development Plan (SDP) for 2020-2024 to continue implementing various reforms. This strategic plan is now the reference tool for training at NPHS during this period. The operational planning of the 2020-2024 SDP is structured chronologically into intervention axes, strategic orientations, effects, products and activities [3].

From the SDP analysis, only one formulated product mentions ICTs in education: “innovative pedagogical strategies, including ICTs, are used.” The plan does not include an axis of intervention or strategic orientation about using ICTs in teaching. However, shortcomings in using ICTs in teaching and learning are clearly mentioned in several situational analysis sections of the SDP. Of the 226 activities listed, no activity is dedicated explicitly to ICT use in education [3]. According to the SDP designers, three activities are related to pedagogical innovation, integrating the use of ICTs. These activities specifically concern the development of audiovisual teaching-learning tools, the reinforcement of the capacities of 200 actors on the use of these tools, and the organization of follow-up trips. Beyond the use of these tools, the training should aim at enabling teachers to design teaching materials and produce multimedia courses [34]. One activity concerns the construction of multimedia computer rooms for teachers and students. Another, much more global activity relating to infrastructure maintenance, equipment, and logistics is included in the plan.

Moreover, the SDP does not explicitly provide specific and adequate financial resources related to ICT use in education. These weaknesses demonstrate that ICT use in education does not yet seem to be well understood and is insufficiently implemented. To promote ICT use in education, priority actions are performed according to the weaknesses and challenges identified. In particular, the institutional, personal, and pedagogical factors favoring ICT use in education should be emphasized [8,32,33]. To this end, the NPHS should first include in the SDP at its midterm review a specific intervention strategy or effect with relevant activities related to ICT use in education. The school should then develop a specific plan for ICT use in education [8] with input from experts. Finally, the regional directorate should identify the feasible activities of the plan.

In the systematic review, solutions such as the development of appropriate institutional strategies, the motivation of the main actors, the mobilization of financial resources, and the strengthening of infrastructure and equipment were proposed to overcome difficulties [8,32,33]. However, these solutions have not been broken down into activities that can be easily implemented.

### Limitations of the Study and Future Research

This paper has some limitations. It includes two systematic reviews that were conducted quickly because of a time constraint. The systematic review on barriers and facilitators of ICT use in higher education had a sample of 3 articles because a limited number of articles met the criteria. Nonetheless, these articles reported results from a significant number of primary articles. Detailed results on the barriers to ICT use were found in the included articles. However, the results regarding the factors facilitating ICT use were general and sparse. This insufficiency of detailed and abundant results on the strategies to be implemented to overcome the difficulties requires the realization of additional primary research.

Moreover, the lack of use of specific methodologies in the articles to describe the use of ICTs during the COVID-19 pandemic in education shows that the results must be interpreted while taking the limitations of the studies into account. For the two systematic reviews, no grey literature search was performed. Relevant studies may have gone unnoticed.

### Conclusion

Inadequate quality of training, ongoing reforms at the NPHS, and restrictive measures imposed following the advent of the COVID-19 pandemic indicate the need to promote ICTs in teaching and learning. This promotion should be achieved progressively through rigorous planning and according to available resources. Priority actions should focus on institutional, personal, and pedagogical factors that promote ICT use in education. In-depth knowledge of the use or integration of ICTs in teaching-learning by the institution’s officers, teachers, and students and the upgrading of equipment will be essential steps toward the optimal exploitation of ICTs in education at the Burkina Faso NPHS.

## Abbreviations

BMD: Bachelor-Master-Doctorate
DHEHS: Directorate of Higher Education in Health Science
ECOWAS: Economic Community of West African States
ICT: information and communication technology
IT: information technology
NPHS: National Public Health School
PRISMA-P: Preferred Reporting Items for Systematic Review and Meta-Analysis Protocols
SDP: strategic development plan
